# Utilization of an Industry Byproduct, *Corymbia maculata* Leaves, by *Aspergillus terreus* to Produce Lovastatin

**DOI:** 10.3390/bioengineering7030101

**Published:** 2020-08-30

**Authors:** Mishal Subhan, Rani Faryal, Ian Macreadie

**Affiliations:** 1 Department of Microbiology, Faculty of Biological Sciences, Quaid-i-Azam University, Islamabad 45320, Pakistan; mishalsubhan@gmail.com (M.S.); ranifaryal@gmail.com (R.F.); 2 School of Science, RMIT University, Bundoora, VIC 3083, Australia

**Keywords:** *Aspergillus terreus*, eucalyptus, fermentation, lovastatin, SmF, SSF

## Abstract

Due to its ability to lower cholesterol levels, simvastatin is a leading drug for the prevention of strokes and heart disease: it also lowers the incidence of neurodegenerative diseases. Simvastatin is made from lovastatin, a precursor produced by the industrial fungus, *Aspergillus terreus*. In this study, *Corymbia maculata* leaves were tested as a novel substrate for the growth of a new isolate of *A. terreus* and a lovastatin-resistant strain of *A. terreus* to produce lovastatin. *Corymbia maculata* (spotted gum) is well utilized by forest industries as a source of timber because of its high strength, durability and smooth texture. However, the leaves are a major waste product. Growth of *A. terreus* on *Corymbia maculata* leaves, in solid-state fermentation resulted in the production of lovastatin. Fermentation of media using fresh leaves of *Corymbia maculata* produced more lovastatin (4.9 mg g^−1^), than the sun-dried leaves (3.2 mg g^−1^). Levels of lovastatin were further increased by the lovastatin-resistant strain of *A. terreus* (Lvs-r), which produced twice the amount of the parental strain. The production of lovastatin was confirmed by HPLC and LC–MS/MS studies. The study suggests that the utilization of a cheap substrate for the production of lovastatin can have a potential economic benefit.

## 1. Introduction

Forestry involving indigenous species is a major industry in Australia. The timber of *Corymbia maculata* is very strong and is exploited commercially for a range of construction, including outdoor and indoor construction, because of its natural resistance to rot, decay and wood-boring insects. The trees are also used for honey production. The annual wood production potential is 21–35 m^3^ per hectare. However little data are available regarding the waste estimation of the *Corymbia maculata* leaves. There are studies on their use as a biofuel energy source and composting and vermicomposting processes [1,2]. In this study, we have explored the use of *Corymbia maculata* leaves for fungal production of lovastatin.

Lovastatin was the first statin to be approved by the US FDA for the lowering of cholesterol levels [3]. Lovastatin acts as a competitive inhibitor of a rate-limiting enzyme, HMG–CoA reductase (HMGCR) [4]. This enzyme is responsible for the synthesis of mevalonate, which is used in pathways leading to cholesterol production in humans and ergosterol production in fungi [5]. Lovastatin and its derivative simvastatin remain important drugs for preventing cardiovascular diseases and neurodegenerative diseases such as Alzheimer’s Disease [6]. A polyketide pathway of *Aspergillus terreus* is used to produce lovastatin and related compounds; these compounds may inhibit the fungal growth. However, fungal resistance to such compounds may lead to higher production of lovastatin, as occurs with other antibiotic-producing industrial strains. Secondary metabolites such as lovastatin are produced after extended growth on complex substrates such as orange peels and rags [7].

**Figure 1:**
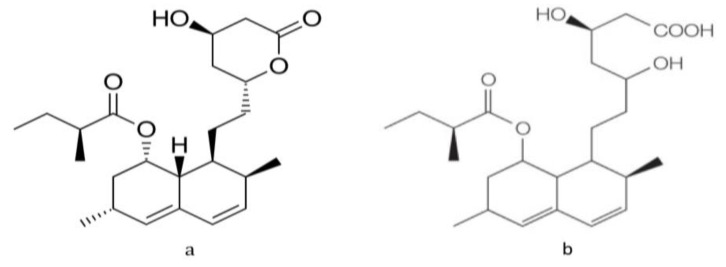
Structure of lovastatin. (**a**) Inactive lactone form (**b**) activated hydroxyl acid form.

The chemical structure of lovastatin in closed-ring lactone and open-ring hydroxyl activated forms are shown in Figure 1.

Lovastatin and simvastatin are taken in the inactive lactone form, which is activated in the liver to produce the active form. In fungal production, lovastatin is released outside the cell as the active beta hydroxyl form—possibly as a defense mechanism—as a secondary metabolite. Solvent extraction converts the active hydroxyl form into the inactive lactone form, which requires treatment with sodium hydroxide ethanolic solution, as these statins exhibit a pH-dependent reaction resulting in hydrolysis of the lactone ring in an alkaline medium [7].

*Aspergillus terreus* is the most commonly used fungus in the industrial production of lovastatin. It is generally cultured in batch and fed-batch fermentation mode at 28–30 °C with a pH around 6.5. The biosynthetic pathway leading to lovastatin production is complex. *A. terreus* possesses a set of gene clusters involved in the biosynthesis of lovastatin through a number of intermediates. The production includes joining of two polyketides by a polyketide synthase system (PKS). This PKS further comprises two domains—the lovastatin nonaketide synthase (LNKS) and lovastatin diketide synthase (LDKS). A number of enzymes participate in tailoring of post-PKS steps leading to the final structure of lovastatin [7,8]. The production of lovastatin is also not limited to *Aspergillus* spp.: it has been reported in several fungal species, such as *Monascus* spp., *Penicillium* spp. including *Penicillium citrinum*, *Penicillium purpurogenum, Paecilomyces* spp., *Pleurotus* sp. and *Trichoderma viride* [9,10].

Commercial production of lovastatin is highly desirable, due to its high demand. The utilization of inexpensive substrates, and readily available renewable natural resources are highly desirable. Furthermore, useful are strategies to overproduce lovastatin, such as through selection of mutant strains that have resistance to feedback inhibition which can limit production. Such strategies can be used to produce lovastatin at higher levels and not only decrease the cost of lovastatin production, but also provide new commercial opportunities [11,12]. The present study includes the use of *Corymbia maculata* leaves as a novel substrate for the production of lovastatin from a wild-type isolate and a lovastatin-resistant mutant (Lvs-r) strain.

## 2. Materials and Methods

### 2.1. Microorganism

The *A. terreus* strain used in this study was isolated from agricultural soil from Pakistan and was characterized using morphologic and molecular 18S rDNA (ITS) analyses. The strain was maintained on Sabouraud dextrose agar (SDA) slants containing 1.5% (*w/v*) agar, 4% (*w/v*) dextrose and 1% (*w/v*) mycological peptone at 4 °C [13].

### 2.2. Production of Lovastatin

A modified soybean-meal medium was used for the evaluation of lovastatin production by *A. terreus*. Modified soybean-meal medium was used as a screening medium: 5% (*w/v*) lactose, 2% (*w/v*) soybean meal, 0.1% (*w/v*) K_2_HPO_4_, 0.1% (*w/v*) NaNO_3_ and 0.05% (*w/v*) MgSO_4_·7H_2_O [14]. Spore suspensions from *A. terreus* were prepared from actively growing slants in sterile saline water, and spores were counted using a hemocytometer. Spores were diluted to 5 × 10^6^ mL^−1^. Each Erlenmeyer flask (250 mL) was inoculated with a spore suspension of 5 × 10^6^ spores mL^−1^ containing 200 mL of soybean-meal medium. The cultures were subjected to fermentation in a shaker incubator at 30 °C and 150 rpm for 12 days. For solid-state fermentation (SSF) 1.5% agar was used to solidify the soybean-meal medium. The preinoculum was prepared by growing cultures of *A. terreus* initially on SDA plates for 2 days by the point inoculation method. Finally, the plates were inoculated with a 1-cm^3^ chunk of agar with growing *A. terreus* and incubated at 30 °C for 12 days.

### 2.3. Downstream Processing

At the end of 12th day of fermentation, the pH of the broth was lowered to 2 with 1-N HCl. An equal volume of ethyl acetate (EtOAc) was added in each flask and incubated at room temperature. The fermentation samples were subsequently centrifuged at 3354 g for 10 min. The organic layer on the top was separated, and the lower aqueous was filtered through a separating funnel. In the case of SSF the agar media in plates was finely ground and the material was dissolved with an equal amount of EtOAc and incubated overnight. The organic layer was separated and dried using a rotary vacuum evaporator. The dried residues were dissolved in acetonitrile. The crude extracts obtained for each sample were used for qualitative and quantitative analyses [14].

### 2.4. HPLC

HPLC was carried out using a reverse phase C-18 Hypersil column (5-μm × 4-mm × 125-mm-long) with an Agilent technologies 1220 LC. A pure standard of lovastatin was purchased from Sigma-Aldrich. The stock standard of lovastatin was 500 µg mL^−1^ in 100% acetonitrile. Acetonitrile was used with water in a ratio of 60:40 in an isocratic system acidified with 0.1% orthophosphoric acid as a mobile phase. The peaks were analyzed at 238 nm. The flow rate was kept constant as 1.5 mL min^−1^ with the injection volume of 20 µL [10]. HPLC of lovastatin at various concentrations was used to derive a standard curve, and this was used to quantify the amounts of lovastatin produced under various conditions.

### 2.5. LC–MS

Mass spectra of crude extracts were analyzed by an Agilent 6410A triple Quadrupole LC/MS system coupled to an Agilent 1200 series rapid resolution system. The positive ion spray method was used as an ESI+ ion source. An Agilent ZORBAX Eclipse Plus C18 column (1.8 μm particles, 2.1 mm × 50-mm-long) was used for LC/MS analysis. The mobile phase was a gradient system with A containing water with 0.1% formic acid and B consisting of methanol with 0.1% formic acid. The LC system was operated at a flow rate of 500 μL min^−1^ and column temperature of 45 °C. The column eluent was introduced into the electrospray ionization source. The nebulizing gas flow-rate was 11 mL/min, drying gas temperature was 325 °C, with the capillary voltage at 4000 V. The injection volume was 4 μL. The fragmentor voltage was kept at 100 V and the collision energy was 7 V. The MS range was kept at 100 to 2000 *m/z*. The MRM (multiple reaction monitoring) analysis mode was used for the confirmation of compounds in the crude extracts.

### 2.6. Quantification of Lovastatin in Samples

Dilutions of the lovastatin stock were analyzed to derive a standard curve. All the crude extract samples were also dissolved in 100% acetonitrile and analyzed by HPLC for final quantitation.

### 2.7. Optimization of Carbon and Nitrogen Sources

The carbon and nitrogen sources were optimized to achieve high titers of lovastatin. Different carbon sources with levels ranging from 1–5% (*w/v*) were tested. The carbon sources that were used included starch, sucrose, mannitol, cellulose, carboxymethyl cellulose (CMC), galactose, dextrose, lactose, xylose, maltose and polyethylene glycol 3350 (PEG). Different nitrogen sources were also used for the optimization study with levels ranging from 1–5% (*w/v*). The nitrogen sources included peptone, yeast extract, corn meal and soybean oil. The fermentation was done as solid-state fermentation (SSF).

### 2.8. Fermentation of Eucalypt Leaves as a Carbon Source

Sun-dried leaves and fresh leaves from *Corymbia maculata* (spotted gum) were collected and reduced to a fine powder in a blender. The solidified soybean meal media and the lactose were replaced with different concentrations of the eucalyptus leaves (1%, 3% and 5%) in the medium. The preinoculum was prepared by growing cultures of *A. terreus* on SDA plates for 2 days by the point inoculation method. Finally, the plates were inoculated with a 1 cm^3^ chunk of agar with growing *A. terreus* and incubated at 30 °C for 12 days. Extraction was done using EtOAc as a solvent.

### 2.9. Resistance to Lovastatin and Production of Lovastatin by Over-Producer

The wild-type strain of *A. terreus* was grown in a flask inoculated with a concentration of 1 mM preactivated lovastatin and incubated at 30 °C with shaking at 150 rpm for 15 days. The strain was also inoculated on SDA plates supplemented with different concentrations of preactivated lovastatin ranging from 10 μM–1500 μM. The spontaneous lovastatin mutant (Lvs-r) was able to grow in the presence of 1 mM of lovastatin, while wild-type was unable to grow in lovastatin levels greater than 50 μM.

The wild-type and Lvs-r strains were inoculated separately on plates containing 3% eucalyptus leaves a carbon source. Plates were incubated at 30 °C for 12 days. After incubation plates were prepared for solvent extraction. The extraction involved the crushing of solidified media and EtOAc solvent being added three times to extract the compounds. The biomass sedimented down and the upper layer of solvent was extracted and solvent was dried using a rotary vacuum evaporator.

### 2.10. Effects of Solvents on Lovastatin Extraction

Downstream processing was carried out using EtOAc as an extraction solvent. Methanol and chloroform were also analyzed for extractions which were evaluated using HPLC.

### 2.11. Statistical Analysis

Statistical analysis was performed for all experiments and done in triplicates. Graphs were generated and standard deviation were calculated using Microsoft Office Excel 2010 software and PRISM 7 version 7.0b for Mac OS X (GraphPad Software, Inc., La Jolla, CA, USA).

## 3. Results

### 3.1. HPLC Analysis

**Figure 2:**
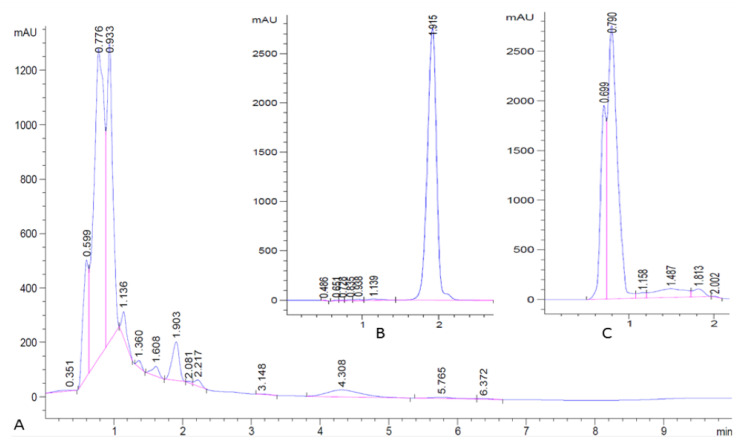
HPLC chromatogram. (**A**) Crude extract from *Aspergillus terreus*; (**B**) pure lovastatin standard; (**C**) blank.

Samples of pure lovastatin and EtOAc extracts of *A. terreus* cultures were analyzed by HPLC. EtOAc extracts from *A. terreus* cultures had a UV absorbing peak eluting with a retention time (1.9 min) identical to pure lovastatin as shown in Figure 2A,B. Further, EtOAc extracts of uninoculated culture media, just contained the media substrates and did not yield UV-absorbing material with this retention time (Figure 2C). LC–MS confirmed that this peak was solely composed of lovastatin.

### 3.2. Optimization of Carbon and Nitrogen Sources

High levels of lovastatin were achieved by optimization of carbon and nitrogen sources. HPLC was used for the quantification and yields were calculated as mg g^−1^ of substrate.

**Figure 3:**
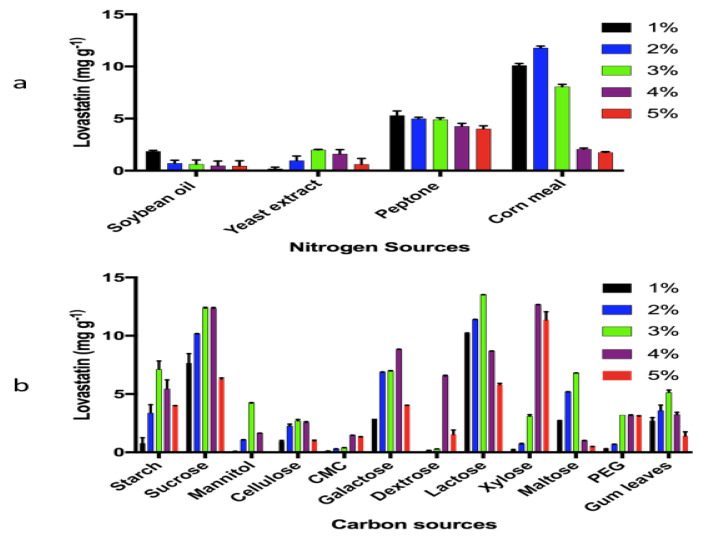
Optimization of (**a**) nitrogen sources and (**b**) carbon sources.

Maximum yields of lovastatin were most often associated with carbon sources at 3% and 4%. The highest level of lovastatin production, 13.5 mg g^−1^, was achieved with 3% lactose (Figure 3b). In the case of nitrogen optimization, most N sources of 1–2% resulted in the greatest production lovastatin. The maximum lovastatin production, 11.7 mg g^−1^, was achieved using 2% corn meal as the nitrogen source (Figure 3a).

### 3.3. Production of Lovastatin with Corymbia maculata Leaves as a Carbon Source

Recent research has evaluated the potential of eucalyptus leaves as source for biofuel and other biological products [15,16,17]. Eucalyptus leaves are very fibrous and low in protein nutrition and were tested as a substrate for lovastatin production. Fresh and dried leaves of the spotted gum (*Corymbia maculata*), a tree species that is widespread and abundant in Australia, were tested for their effect on production of lovastatin by *A. terreus*. *A. terreus* grew well on both fresh and dried leaves. The fresh leaves at 3% (*w/v*) resulted in the maximum production of 4.9 mg g^−1^ while dried leaves led to levels of 3.2 mg g^−1^ of lovastatin as judged by HPLC quantitation. HPLC analysis of extracts from uninoculated media showed no absorbance corresponding to the pure lovastatin standard (data not shown).

### 3.4. Production of Lovastatin by a Lovastatin-Resistant (Lvs-r) Strain

**Figure 4:**
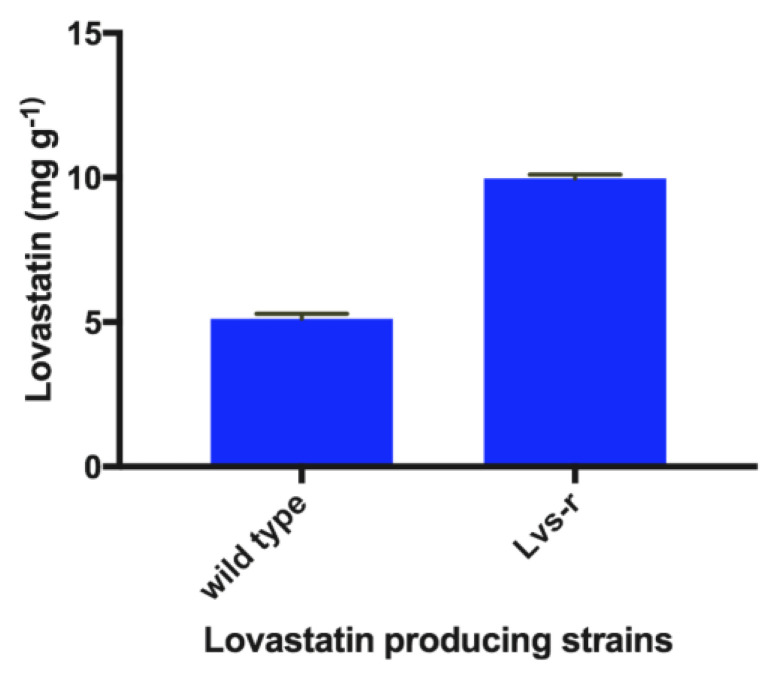
Lovastatin production in the wild-type and lovastatin-resistant strains (Lvs-r).

After 12 days of culture incubation, plates inoculated with the original and the resistant Lvs-r *A. terreus* strains were extracted with EtOAc. Levels of lovastatin produced were quantified by HPLC. The amount of lovastatin produced using eucalyptus leaves as carbon source by the Lvs-r strain was twice (10.1 mg g^−1^) the amount produced by the original strain (4.9 mg g^−1^), as shown in Figure 4.

### 3.5. LC–MS Analysis

Detection of statins in biologic samples like blood and crude extracts can readily be done by LC–MS and MS–MS methods. These methods are sensitive, precise and highly selective for the purpose of studying statins, whether in biologic samples or crude extracts that were utilized in our study [18,19].

**Figure 5:**
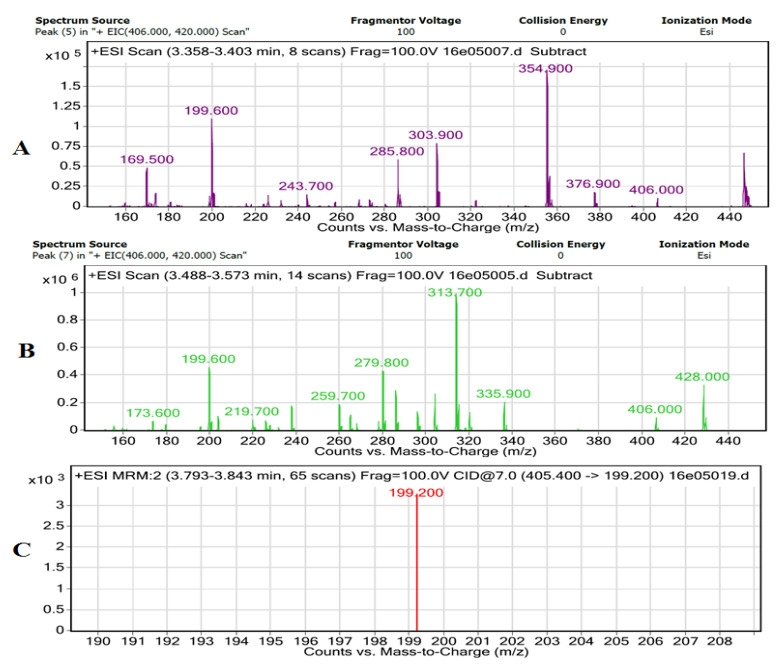
Mass spectra analyses. (**A**) LC–MS + ESI scan of lovastatin with lactose as a C-source; (**B**) LC–MS + ESI scan of lovastatin with eucalyptus leaves as a C-source; (**C**) MS–MS ion mass spectra of lovastatin with eucalyptus leaves as a C-source.

To further confirm the presence of lovastatin produced by *A. terreus* during the fermentation of the leaves, LC–MS–MS analysis was performed. This analysis is shown in Figure 5. The spectrum (Figure 5A,B) indicates the presence of lovastatin in the sample. The ion mass spectrum of analyte is shown in Figure 5C. The most abundant product ion was selected for MRM monitoring. The protonated molecule [M + H] + was monitored in the electrospray positive ionization mode. Therefore, the ion transition *m/z* 405.0 > 199.0 was selected for MRM of the lovastatin. Argon was used as a collision gas and collision energy was optimized for the analyte. The stereochemistry of lactone ring also effects the dehydration of lactone in lovastatin. In the current study, we did not investigate whether the lovastatin was in the closed lactone or B-hydroxyl open-ring structure, but the ion at *m/z* 199 is reported to be independent of the functional groups (ester and OH) of the lactone moiety as consistent with current study, which confirms the presence of lovastatin in the sample [20].

## 4. Discussion

The production of lovastatin by *Aspergillus terreus* is important for pharmaceutical production. Lovastatin is chemically modified to produce simvastatin, an important drug for the prevention of CVD and neurodegenerative disease. In any fermentation system the components of the culture medium have a direct influence on the production of metabolites. Among them carbon and nitrogen sources play a major role as these nutrients are directly related to the synthesis of metabolites and biomass during fermentation [7,9]. The polyketide pathway through which lovastatin is synthesized utilizes carbon sources more slowly than the pathway leading to production of biomass. Use of slowly metabolizing disaccharides such as lactose enhances the production of lovastatin [21,22]. The metabolic pathway leading to the production of lovastatin is greatly enhanced by nitrogen source limitation. By limiting the nitrogen source, the excess carbon source is utilized by *A. terreus* to synthesize secondary metabolites, thus increasing the yield of lovastatin [7].

In this study, the use of *Corymbia maculata* leaves was shown for the first time to our knowledge, to be a substrate for growth of *A. terreus* and to lead to reasonable levels of lovastatin. Green leaves led to the production of more lovastatin than dried leaves. This may be a consequence of the higher moisture content in fresh leaves. However, it may also be due to higher levels of bioactive compounds in the fresh leaves. Extracts from the eucalyptus comprise more than 100 bioactive compounds such as saponins, steroids, glycosides, tannins, volatile oils and phenols [23]. There are several studies on the production of lovastatin on SSF, using alternate growth media such as agroindustrial residues and waste. Rice straw led to production of lovastatin at levels of 0.26 mg g^−1^ wheat bran led to 3.723 mg g^−1^, rice powder led to 2.9 mg g^−1^ and coconut oil cake led to 7.43 mg g^−1^ under SSF conditions by *Aspergillus* spp. [7]. To our knowledge, this is the first time *C. maculata* was reported as a growth medium to produce lovastatin. Lovastatin production is known to be increased by growth on substrates that are challenging, and eucalypt leaves appear to be a further example. A further advantage of these leaves is that they are largely a waste product of the forestry industry, so their utilization adds value to that industry. *Corymbia maculata* is just one of hundreds of species of eucalypt trees: it is worth investigating if other eucalypt leaves could offer the same benefits.

A comparative analysis of solid-state fermentation (SSF) and submerged fermentation (SmF) was evaluated for production of lovastatin from *A. terreus*. SSF can be a better alternative due to easier downstream processing and higher yields with economical and low cost production [24,25]. With identical media the amount of lovastatin produced by SSF (2.6 mg g^−1^), was double that produced by SmF (1.3 mg g^−1^) before optimization, in agreement with other studies [26,27]. Several studies have reported SSF to be preferred for fermentation in comparison to SmF as it is more cost-effective and increases the yield of the metabolites [28,29]. The US FDA has approved SSF as a preferred process of fermentation for production of medicinal drugs of fungal origin [29].

Recovery of lovastatin from *A. terreus* is also an important factor that contributes to costs. Three solvents, EtOAc, methanol and chloroform were compared to determine the best solvent for lovastatin extraction. The highest recoveries of lovastatin were achieved with extraction by EtOAc. chloroform and methanol led to better separation of various other compounds in comparison to EtOAc, however, that is of no consequence to the current study. EtOAc has been reported to increase the sensitivity of detection and solubility of lovastatin due to its semi-polar nature [30]. Methanol did not result in higher levels of lovastatin, due to the fact that it results in further transformation of the lactone to the methyl ester form, thus contributing to the structural instability [7,31]. Extraction of lovastatin decreased with the gradual decrease in polarity of the organic solvents used [32]. Other studies have also sought to optimize the solvent for the maximum recovery of lovastatin [32,33].

*Aspergillus terreus* is inhibited by lovastatin so the isolation of a lovastatin-resistant strain was examined. Production of lovastatin in the resistant strain was double that of the parent strain, indicating product inhibition is a major limit on lovastatin production. It would be of interest to determine whether even higher levels of resistance could be selected and lead to higher levels of lovastatin production. There is little information on the basic underlying mechanism of statin resistance and production of lovastatin by lovastatin-resistant mutants. The only study reported so far on statin resistance is reported in yeast cells. *Candida glabrata* appeared to gain resistance by overexpression of HMGCR, the target of statins. Statins inhibit *C. glabrata* by reducing ergosterol (the yeast equivalent of cholesterol) levels, but this inhibition can be overcome by producing more HMGCR [34].

## 5. Conclusions

This work investigated some culture and extraction parameters to optimize production and analyze the hyper-production and recovery of lovastatin, which remains an important drug, that is nowadays chemically converted to simvastatin for prevention of cardiovascular and neurodegenerative diseases. The use of a *Corymbia maculata* is a novel contribution and suggests that it could be worthwhile to investigate other abundant Australian native tree leaves as a carbon-sources for production of secondary metabolites.
